# Patient-Perceived feasibility of implementing OpenCap smartphone-based motion capture technology to remotely assess musculoskeletal movement in individuals with orthopedic disorders presenting to a rural midwestern tertiary center

**DOI:** 10.3389/fdgth.2026.1870758

**Published:** 2026-07-23

**Authors:** Holly D. Aitken, Joshua B. Holt, Robert W. Westermann, Jacob M. Elkins, Dallas A. Vanorny, Justin T. Crawmer, Eli S. Gregory, Silvana Velasquez-Marin Mohr, Cole M. Rich, Jessica E. Goetz

**Affiliations:** 1 Department of Orthopedics & Rehabilitation, University of Iowa Health Care, North Liberty, IA, United States; 2 Department of Biomedical Engineering, University of Iowa, Iowa City, IA, United States

**Keywords:** motion capture, OpenCap, orthopedics, remote evaluation, technological access

## Abstract

**Background:**

OpenCap, an open-source research platform for estimating 3D joint kinematics from videos captured with two iOS devices, has the potential to enable remote rehabilitation tracking and reduce travel burden associated with in-person orthopedic evaluation. However, it is unknown whether orthopedic patients have adequate iOS technological access required for OpenCap data collection, particularly in rural or resource-limited settings in which getting to clinic for rehabilitation management is most difficult. This study sought to evaluate orthopedic patient access to the necessary technology and perceived comfort level with performing an OpenCap data collection.

**Methods:**

Three orthopedic patient cohorts were prospectively enrolled: (1) 200 pediatric patients (and their caregivers), (2) 200 adult sports medicine patients, and (3) 200 adult joint arthroplasty patients. Participants completed a REDCap questionnaire regarding their access to a computer, internet, and iOS devices, and their perceived comfort level performing a remote musculoskeletal biomechanics assessment.

**Results:**

Of the orthopedic patients enrolled, 65.5–83.5% indicated access to the necessary technology resources for an OpenCap data collection, and geographic residential location did not significantly affect technological access. 71.7%–95.8% of patients indicated that they were “definitely” or “probably” comfortable performing activities needed for an OpenCap data collection, and 68.3%–82.3% of patients felt that the OpenCap technology would “definitely” or “probably” reduce their travel burden associated with in-person clinical assessment.

**Conclusions:**

While both kinematic and implementation validation in specific orthopedic patient populations is still required, these findings suggest the perceived feasibility of using OpenCap for future remote musculoskeletal biomechanics assessment among orthopedic patients, including those in predominantly rural settings.

## Introduction

Diagnosing, treating, and evaluating recovery from orthopedic conditions ubiquitously involves assessing a patient's limb or joint movement (1–4). Repetitive travel for rehabilitation can be especially challenging for mobility-limited orthopedic patients, particularly those in remote rural locations. Recent advancements in automated video analysis have enabled development of novel tools that can provide objectively quantified human movement data (5–9). Integration of such technology could shift the paradigm of clinical practice, in which patients must travel to be seen at a medical facility where clinicians assess movement relying primarily on qualitative, subjective evaluations.

OpenCap (Stanford, USA) is one such tool that utilizes automated, markerless motion capture for musculoskeletal biomechanics assessment. It uses open-source pose estimation (10–14) to detect two-dimensional (2D) landmarks on a subject from synchronized video recordings. The landmarks are reconstructed into three-dimensional (3D) landmarks (15, 16) and used to predict 3D anatomical marker positions (17) for scaling a baseline musculoskeletal model (17–19) to the subject's anthropometry. This personalized musculoskeletal model is then used to calculate 3D joint angles (20) during the subject's movement. While this process of obtaining 3D joint kinematics from 2D videos is complex, it is a fully automated, cloud-based process—users simply record and upload synchronous videos and then can view clinically relevant motion analysis through OpenCap's web interface (21).

Collecting and analyzing movement data using OpenCap requires two iOS devices for video capture and a laptop/computer to access OpenCap's web application (21). This starkly contrasts with the specialized cameras and equipment required by traditional marker-based systems and holds potential to minimize the user variability and accessibility limitations of laboratory-based motion capture environments (22–25). Initial validation of joint kinematics derived from the two-camera OpenCap technology compared to marker-based analysis for walking, squatting, chair rise, and drop jumps in 10 healthy individuals demonstrated mean absolute errors of 1.7–10.3° (21). Subsequent studies have expanded validation for other functional tasks (26–28) and in the context of certain pathologic conditions (29–32), demonstrating OpenCap's broad validity for musculoskeletal biomechanics assessment.

While OpenCap is fundamentally an open-source research platform, it has the potential to provide orthopedic clinicians with quantitative movement data and to reduce travel burden associated with in-person evaluation for orthopedic patients. However, the dependence of OpenCap on multiple iOS devices specifically may limit its widespread use outside of research institutions, particularly in rural or resource-limited settings in which implementing such technology for remote rehabilitation tracking would be most beneficial (33–35). The technological burden on the patient to use a primarily research-focused software themselves, particularly in older patients, may also prevent its widespread implementation (36, 37). Therefore, the objective of this study was to determine access to the necessary physical/digital technology and theoretical comfort level with performing activities required for an OpenCap data collection in patients presenting for orthopedic care in a predominantly rural state. We hypothesized that patients in our eldest cohort (joint arthroplasty patients) would have the least access to the required technology and be least comfortable with the idea of using that technology to measure their movement.

## Methods

Under Institutional Review Board approval, three patient cohorts were prospectively enrolled between May 1st and November 30th, 2025 at our institution: (1) 200 pediatric patients of adolescent age (ages 10–17 years) being treated/monitored by a pediatric orthopedic provider (plus their caregivers), (2) 200 adult patients (ages 18–50 years) being treated/monitored by a sports medicine orthopedic provider, and (3) 200 older adult patients (ages 51–99 years) being treated/monitored by a total joint arthroplasty orthopedic provider. These three patient cohorts encompass distinct life stages and generational differences that likely influence their previous technological experience, plus a broad range of distinct orthopedic conditions, thereby best representing the general orthopedic patient community presenting to a tertiary care center in a predominantly rural state. Patients who were unable to provide consent due to neuromuscular or neurodevelopmental conditions were excluded.

**Figure 1:**
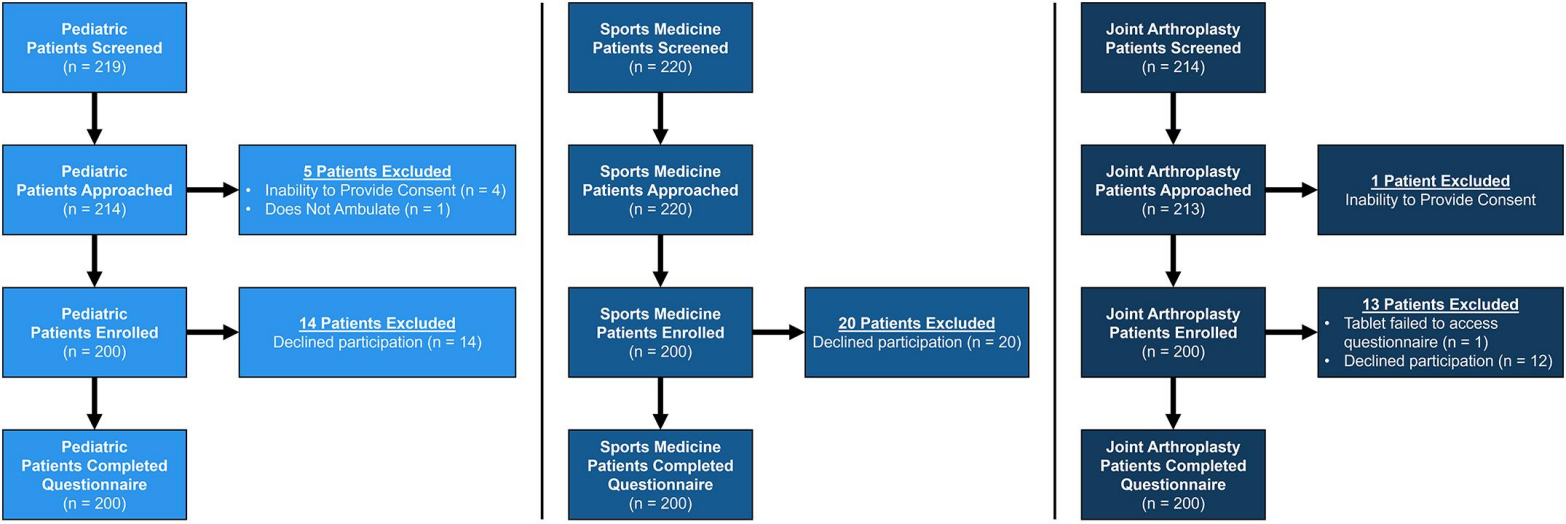
Patient recruitment flow diagram for pediatric (left, light blue), sports medicine (middle, blue), and joint arthroplasty (right, dark blue) patients.

Study participants were identified by screening upcoming consecutive orthopedic clinics for eligible patients, and all patients meeting inclusion criteria were approached during their orthopedic clinic visit regarding study participation (Figure 1). Upon study enrollment and consent, patients (with caregiver assistance as necessary) were administered the first tier of questions regarding their orthopedic provider, orthopedic condition, annual household income (self-reported as an optional survey item), and technology access perceptions (i.e., perceived ability to have access to two iOS devices simultaneously). Participants who perceived having the necessary technological access were then asked the second tier of questions regarding their perceived technology application comfort level, including their ability to set up the iOS devices in a stationary manner, their comfort level installing an app on the iOS devices, and their simultaneous access to two iOS devices and a computer with internet connection. Participants who perceived having the necessary technological access (tier 1) and being comfortable using an app on the iOS devices (tier 2) were then asked the third tier of questions regarding their perceived comfort with performing OpenCap data collection steps. Free-text explanations for indicated comfort levels were permitted. The number of participants who responded to each tier of questions can be found in Tables 1 and 2, and the complete questionnaire that was developed by the authors is provided in Supplementary Appendix A. Patient responses were collected on a tablet in their clinic exam room using HIPAA-compliant REDCap electronic data capture tools hosted at our institution (38, 39), and caregivers aiding with answering questions were instructed that answers should be provided from the perspective of the patient. Participant residence information was categorized using the rural-urban commuting area (RUCA) codes established by the United States Department of Agriculture Economic Research Service (40, 41) to evaluate patient responses based on rural-urban classification.

**Table 1:** Number of pediatric, sports medicine, and joint arthroplasty patients in each geographic residential location that responded to each tier of questions.

Patient Cohort	Geographic residential location	Tier 1—Technology Access Perceptions	Tier 2—Perceived Technology Application Comfort Level	Tier 3—Perceived Comfort with Performing OpenCap Data Collection Steps
Pediatrics	Metropolitan	110	91	82
	Micropolitan	31	26	24
	Small Town	26	21	18
	Rural Areas	33	29	24
Sports Medicine	Metropolitan	120	95	88
	Micropolitan	28	22	22
	Small Town	19	13	11
	Rural Areas	33	25	24
Joint Arthroplasty	Metropolitan	131	80	57
	Micropolitan	28	24	20
	Small Town	17	13	10
	Rural Areas	24	14	9

The total number of pediatric, sports medicine, and joint arthroplasty patients in each geographic residential location that answered the first tier of questions regarding technological access is provided in the first column (totals match those provided in Table 3). The second column indicates the number of patients in each geographic residential location who perceived having the necessary technological access and answered the second tier of questions regarding their perceived comfort level using an app on the iOS devices. The third column provides the number of patients in each geographic residential location who perceived having the necessary technological access and felt comfortable using an app on the iOS devices and therefore answered the third tier of questions regarding their perceived comfort with performing OpenCap data collection steps.

**Table 2:** Number of pediatric, sports medicine, and joint arthroplasty patients in each self-reported annual household income range that responded to each tier of questions.

Patient Cohort	Annual Household Income Range	Tier 1—Technology Access Perceptions	Tier 2—Perceived Technology Application Comfort Level	Tier 3—Perceived Comfort with Performing OpenCap Data Collection Steps
Pediatrics	<$25,000	7	5	3
	$25,000-$49,999	11	4	4
	$50,000-$74,999	19	12	12
	$75,000-$99,999	16	12	10
	$100,000-$124,999	19	18	15
	$125,000-$149,999	25	23	20
	≥$150,000	56	54	52
Sports Medicine	<$25,000	16	11	10
	$25,000-$49,999	21	13	13
	$50,000-$74,999	19	16	14
	$75,000-$99,999	15	13	12
	$100,000-$124,999	15	12	12
	$125,000-$149,999	10	9	8
	≥$150,000	20	20	17
Joint Arthroplasty	<$25,000	20	10	10
	$25,000-$49,999	23	14	6
	$50,000-$74,999	31	17	13
	$75,000-$99,999	25	18	11
	$100,000-$124,999	22	18	14
	$125,000-$149,999	14	12	11
	≥$150,000	21	19	17

The total number of pediatric, sports medicine, and joint arthroplasty patients in each income range that answered the first tier of questions regarding technological access is provided in the first column (totals match those provided in Table 3). The second column indicates the number of patients in each income range who perceived having the necessary technological access and answered the second tier of questions regarding their perceived comfort level using an app on the iOS devices. The third column provides the number of patients in each income range who perceived having the necessary technological access and felt comfortable using an app on the iOS devices and therefore answered the third tier of questions regarding their perceived comfort with performing OpenCap data collection steps.

Statistical analyses focused on descriptive and univariate comparisons consistent with the feasibility objectives of the study. Distributions of patient demographics, residential RUCA classifications, and questionnaire responses were compared between patient groups using chi-square tests for categorical variables. Assumptions for chi-square testing were evaluated by reviewing expected cell counts; Fisher-Freeman-Halton exact tests were used when assumptions were not met. For continuous variables, normality was assessed using Shapiro–Wilk tests to guide selection of appropriate statistical methods, and due to non-normality, distributions of continuous data were compared between patient groups using Kruskal–Wallis tests. Because normality assessments were used descriptively rather than for formal hypothesis testing, no correction for multiple testing was applied. For each patient cohort, distributions of questionnaire responses were also compared between geographic residential locations using chi-square or Fisher-Freeman-Halton exact tests as appropriate. If significant differences were found, then pairwise chi-square, Fisher's exact or Wilcoxon rank-sum tests with Bonferroni multiple-comparison corrections were used to identify which specific distributions significantly differed. Some questionnaire items, including annual household income, were optional, and missing responses were handled using available (complete-case) data for each analysis without imputation. Free-text explanations were descriptively summarized. Analyses were completed using SAS statistical software version 9.4 (SAS Institute), and *p* < 0.05 was considered significant.

## Results

### Tier 1—technology access perceptions

**Figure 2:**
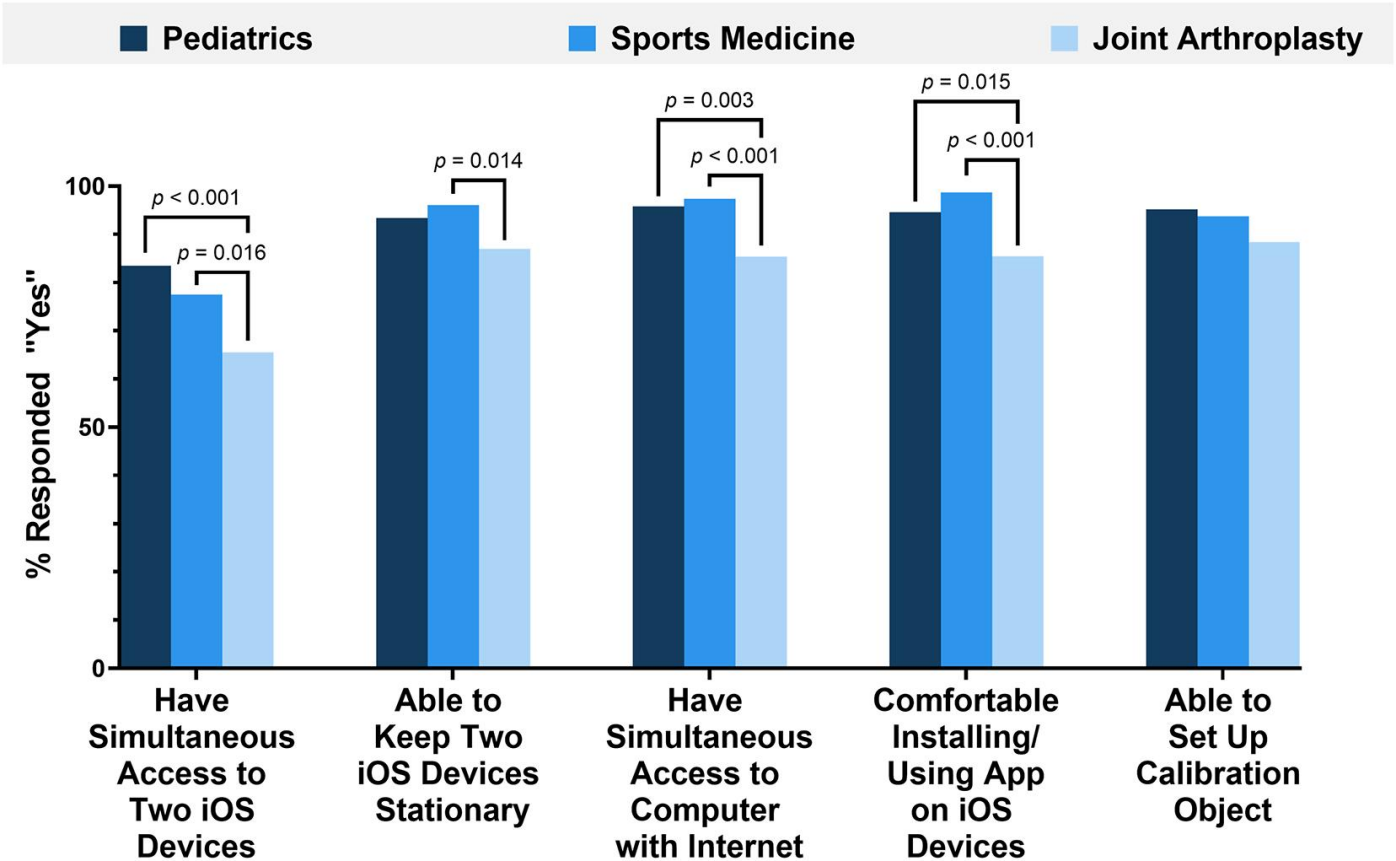
Joint arthroplasty patients indicated less ability to have access to the necessary technological resources and reduced ability to perform specific tasks required for an OpenCap data collection. (First column) Percentage of pediatric (*n* = 200), sports medicine (*n* = 200), and joint arthroplasty (*n* = 200) patients who answered tier 1 questions regarding technology access perceptions. (Second, third, and fourth columns) Percentage of pediatric (*n* = 167), sports medicine (*n* = 155), and joint arthroplasty (*n* = 131) patients who indicated access to two iOS devices and answered tier 2 questions regarding their perceived technology application comfort level. (Fifth column) Percentage of pediatric (*n* = 147), sports medicine (*n* = 144), and joint arthroplasty (*n* = 95) patients who indicated access to two iOS devices, felt comfortable with the technology application, and answered tier 3 questions regarding perceived comfort with performing OpenCap data collection steps.

**Figure 3:**
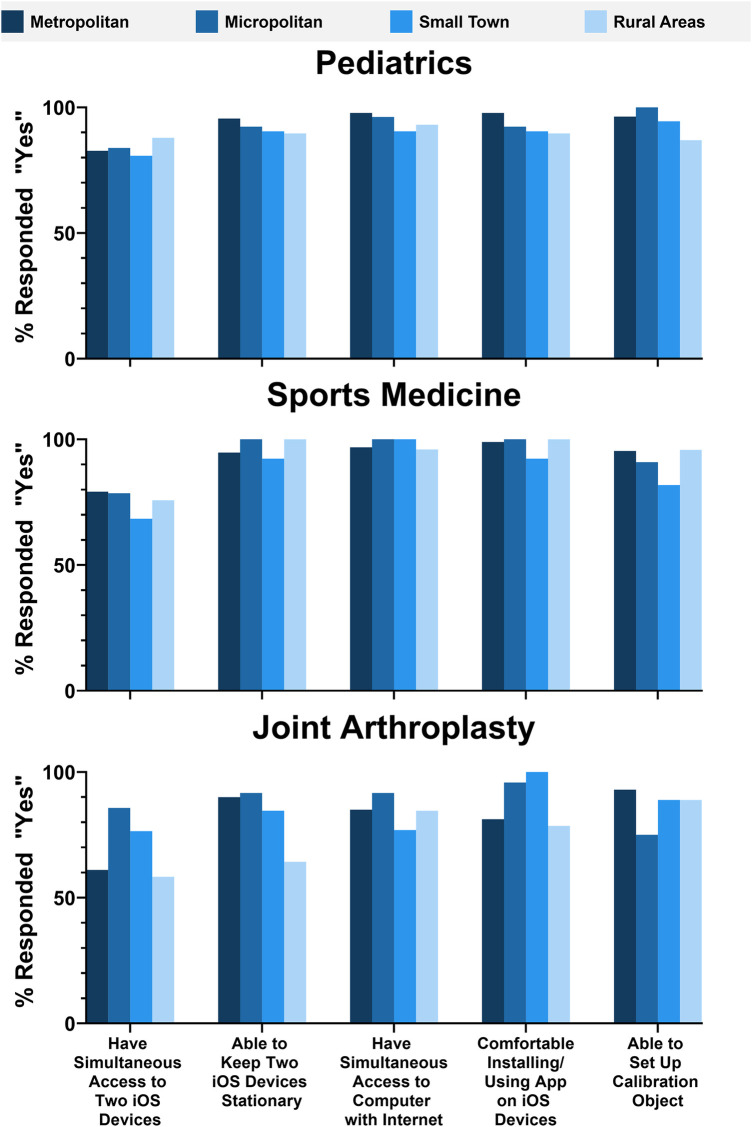
Geographic residential location did not significantly change patient ability to have access to two iOS devices (*p* ≥ 0.091), set up the iOS devices in a stationary manner (*p* ≥ 0.373), access a computer with an internet connection (*p* ≥ 0.212), feel comfortable installing/using an app on the iOS devices (*p* ≥ 0.097), or set up the calibration equipment (*p* ≥ 0.134) for all three patient cohorts. (First column) Percentage of patients in each geographic residential location who answered “Yes” to the first tier of questions regarding technological access. Percentages are out of the total number of patients in each residential location who responded to tier-1 questions (total respondent numbers provided in first column of Table 1). (Second, third, and fourth columns) Percentage of patients in each geographic residential location who indicated having access to two iOS devices and answered “Yes” to the second tier of questions regarding their perceived comfort using an app on the devices. Percentages are out of the total number of patients in each residential location who responded to tier-2 questions (total respondent numbers provided in second column of Table 1). (Fifth column) Percentage of patients in each geographic residential location who felt comfortable using an app on the iOS devices that they believed they could access and who answered “Yes” to the third tier of questions regarding perceived comfort with performing OpenCap data collection steps. Percentages are out of the total number of patients in each residential location who responded to tier-3 questions (total respondent numbers provided in third column of Table 1).

**Figure 4:**
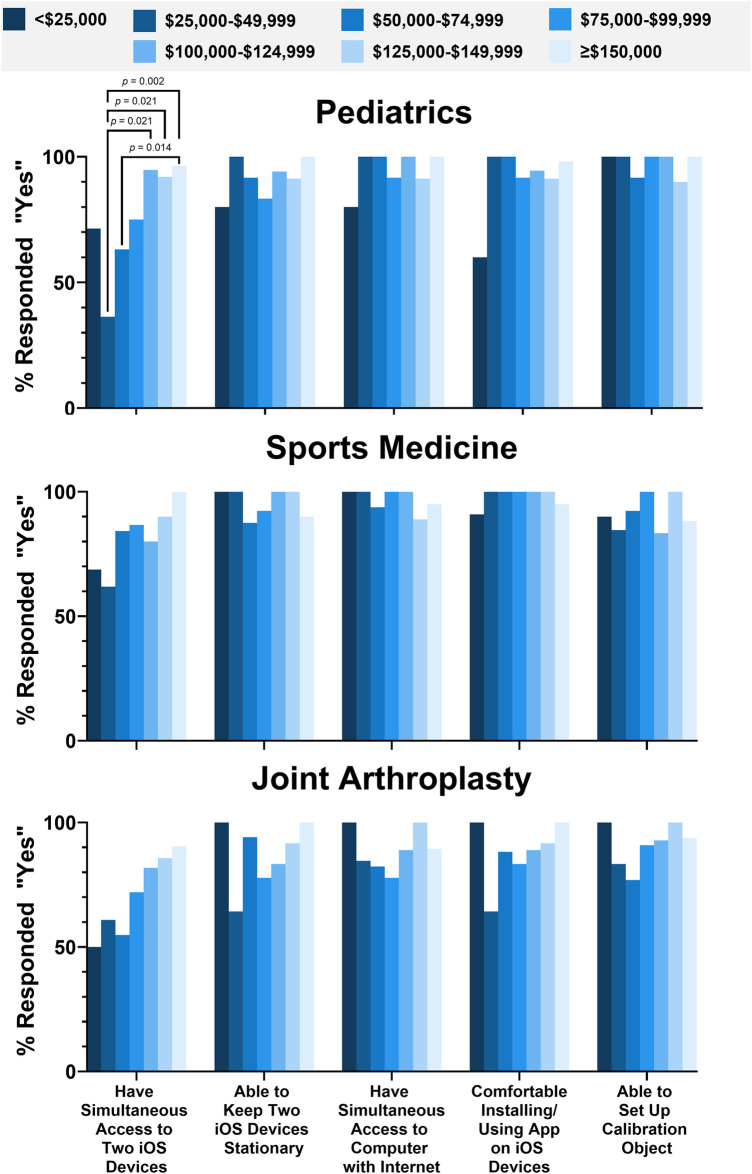
Annual household income only affected pediatric patient ability to have access to two iOS devices (*p* ≤ 0.021). (First column) Percentage of patients in each self-reported annual household income range who answered “Yes” to the first tier of questions regarding technological access. Percentages are out of the total number of patients in each income range who responded to tier-1 questions (total respondent numbers provided in first column of Table 2). (Second, third, and fourth columns) Percentage of patients in each income range who indicated having access to two iOS devices and answered “Yes” to the second tier of questions regarding their perceived comfort using an app on the devices. Percentages are out of the total number of patients in each income range who responded to tier-2 questions (total respondent numbers provided in second column of Table 2). (Fifth column) Percentage of patients in each income range who felt comfortable using an app on the iOS devices that they believed they could access and who answered “Yes” to the third tier of questions regarding perceived comfort with performing OpenCap data collection steps. Percentages are out of the total number of patients in each income range who responded to tier-3 questions (total respondent numbers provided in third column of Table 2).

Patients in each cohort (*n* = 200 in each) were significantly (*p* < 0.001) different in age (by study design) but not gender, race, ethnicity, or RUCA classifications (Table 3). The distribution of annual household income was skewed towards ≥$150,000 in pediatric patients, whereas sports medicine and joint arthroplasty groups demonstrated bimodal distributions with peaks of $25,000-$75,000 and ≥$150,000 (Table 3). 57.5% of pediatric patients were being treated for orthopedic spine conditions, whereas 89.5% and 100% of sports medicine and joint arthroplasty patients, respectively, were being treated for hip and knee orthopedic conditions (Table 3). Fewer joint arthroplasty patients (65.5%) indicated access to two iOS devices simultaneously than both pediatric (83.5%, *p* < 0.001) and sports medicine (77.5%, *p* = 0.016) patients (Figure 2). Residential location (Figure 3) did not significantly change patient-perceived ability to access two iOS devices for any cohort (*p* ≥ 0.091). Annual household income (Figure 4) only significantly affected pediatric patient ability to have access to two iOS devices (*p* ≤ 0.021).

**Table 3:** Comparison of demographic characteristics, annual household income, and orthopedic condition being treated in pediatric, sports medicine, and joint arthroplasty patients.

Patient Demographics	Pediatrics (*n* = 200)	Sports Medicine (*n* = 200)	Joint Arthroplasty (*n* = 200)	*p*-value
Age, years [Median (IQR)]	14.2 [12.5–16]	28.8 [21.8–38.9]	67.7 [59.2–72.8]	<0.001^a^^,^^b^^,^^c^^,^^d^
Female [*n* (%)]	105 [52.5]	127 [63.5]	114 [57]	0.082
Race				0.644
American Indian or Alaska Native [*n* (%)]	1 [0.5]	0 [0]	1 [0.5]	
Asian [*n* (%)]	1 [0.5]	1 [0.5]	4 [2]	
Black or African American [*n* (%)]	8 [4]	7 [3.5]	8 [4]	
* *Native Hawaiian or Other Pacific Islander [*n* (%)]	0 [0]	1 [0.5]	1 [0.5]	
* *Unknown [*n* (%)]	17 [8.5]	15 [7.5]	9 [4.5]	
* *White [*n* (%)]	173 [86.5]	176 [88]	177 [88.5]	
Ethnicity				0.378
* *Hispanic or Latino [*n* (%)]	5 [2.5]	5 [2.5]	1 [0.5]	
* *Non-Hispanic [*n* (%)]	188 [94]	185 [92.5]	193 [96.5]	
* *Unknown [*n* (%)]	7 [3.5]	10 [5]	6 [3]	
Orthopedic Condition Being Treated				<0.001^a^^,^^b^^,^^c^^,^^d^
* *Elbow [*n* (%)]	17 [8.5]	1 [0.5]	0 [0]	
* *Foot/Ankle [*n* (%)]	24 [12]	0 [0]	0 [0]	
* *Hip [*n* (%)]	14 [7]	101 [50.5]	104 [52]	
* *Knee [*n* (%)]	21 [10.5]	82 [41]	99 [49.5]	
* *Shoulder [*n* (%)]	4 [2]	18 [9]	0 [0]	
* *Spine [*n* (%)]	115 [57.5]	1 [0.5]	2 [1]	
* *Wrist/Hand [*n* (%)]	18 [9]	0 [0]	0 [0]	
Residential RUCA^e^ Classification				0.423
* *Metropolitan (RUCA 1–3) [*n* (%)]	110 [55]	120 [60]	131 [65.5]	
* *Micropolitan (RUCA 4–6) [*n* (%)]	31 [15.5]	28 [14]	28 [14]	
* *Small Town (RUCA 7–9) [*n* (%)]	26 [13]	19 [9.5]	17 [8.5]	
* *Rural Areas (RUCA 10) [*n* (%)]	33 [16.5]	33 [16.5]	24 [12]	
Annual Household Income	*n* = 153	*n* = 116	*n* = 156	<0.001^a^^,^^b^^,^^c^
* *<$25,000 [*n* (%)]	7 [4.6]	16 [13.8]	20 [12.8]	
* *$25,000-$49,999 [*n* (%)]	11 [7.2]	21 [18.1]	23 [14.7]	
* *$50,000-$74,999 [*n* (%)]	19 [12.4]	19 [16.4]	31 [19.9]	
* *$75,000-$99,999 [*n* (%)]	16 [10.5]	15 [12.9]	25 [16]	
* *$100,000-$124,999 [*n* (%)]	19 [12.4]	15 [12.9]	22 [14.1]	
* *$125,000-$149,999 [*n* (%)]	25 [16.3]	10 [8.6]	14 [9]	
* *≥$150,000 [*n* (%)]	56 [36.6]	20 [17.2]	21 [13.5]	

a Significant *p*-value comparing pediatric, sports medicine, and joint arthroplasty groups.

b Significant *p*-value comparing pediatric and sports medicine groups.

c Significant *p*-value comparing pediatric and joint arthroplasty groups.

d Significant *p*-value comparing sports medicine and joint arthroplasty groups.

e Rural-urban commuting area (RUCA) codes (40, 41).

### Tier 2—perceived technology application comfort level

Among respondents indicating they would have access to two iOS devices (pediatric *n* = 167, sports medicine *n* = 155, joint arthroplasty *n* = 131) in Tier 1 questions, fewer joint arthroplasty patients (87%) felt that they would be able to set up the iOS devices in a stationary manner than sports medicine patients (96.1%, *p* = 0.014), but this was not different from the pediatric patients (93.4%, *p* = 0.126). Joint arthroplasty patients were less likely to have simultaneous access to a computer with an internet connection (85.4%) or to feel comfortable installing/using an app on the iOS devices (85.5%) than either sports medicine (computer—97.4%, *p* < 0.001; app – 98.7%, *p* < 0.001) or pediatric patients (computer – 95.8%, *p* = 0.003; app – 94.6%, *p* = 0.015). For all three patient cohorts, neither residential location (Figure 3) nor annual household income (Figure 4) significantly affected patient-perceived ability to set up the iOS devices in a stationary manner (residential – *p* ≥ 0.373; income – *p* ≥ 0.176), access a computer with an internet connection (residential – *p* ≥ 0.212; income – *p* ≥ 0.554), or feel comfortable installing/using an app on the iOS devices (residential – *p* ≥ 0.097; income – *p* ≥ 0.059).

### Tier 3—perceived comfort with performing OpenCap data collection steps

Among respondents who indicated being comfortable using an app on iOS devices (Tier 2) that they believed they could access (Tier 1) (pediatrics: *n* = 147; sports medicine: *n* = 144; joint arthroplasty: *n* = 95), there was no difference (*p* = 0.115) among the three patient cohorts (pediatrics: 95.2%; sports medicine: 93.8%; joint arthroplasty: 88.4%) in perceived ability to set up the necessary equipment for OpenCap calibration (Figure 2).

**Figure 5:**
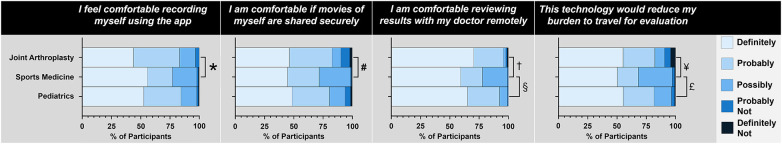
Distributions of pediatric (*n* = 147), sports medicine (*n* = 144), and joint arthroplasty (*n* = 95) patient comfort level performing specific tasks required to use the OpenCap technology and perceived reduction in travel burden associated with in-person evaluation. While significant differences in distributions of patient comfort level and perceived burden reduction do exist between patient groups, 71.7%–95.8% of patients indicated that they were “definitely” or “probably” comfortable with performing activities required for an OpenCap data collection, and 68.3%–82.3% of patients felt that the OpenCap technology would “definitely” or “probably” reduce their burden to travel for in-person clinical assessment. *p = 0.022 between sports medicine and joint arthroplasty patients. ^#^*p* = 0.004 between sports medicine and joint arthroplasty patients.^†^*p* < 0.001 between sports medicine and joint arthroplasty patients. ^§^*p* = 0.002 between pediatric and sports medicine patients.^¥^*p* = 0.002 between sports medicine and joint arthroplasty patients.^£^*p* = 0.019 between sports medicine and joint arthroplasty patients.

84.5% and 83.3% of pediatric and joint arthroplasty patients, respectively, indicated being “definitely” or “probably” comfortable recording themselves walking, squatting, and performing a sit-to-stand task using the OpenCap technology, in comparison to 77.2% of sports medicine patients (Figure 5). Similar trends were demonstrated for patients indicating being “definitely” or “probably” comfortable (1) sharing those video recordings with Stanford University for OpenCap's automated analysis (pediatric: 80.4%, sports medicine: 71.7%, joint arthroplasty: 82.8%) and (2) reviewing results from OpenCap's automated analysis remotely with their doctor (pediatric: 92.5%, sports medicine: 77.9%, joint arthroplasty: 95.8%). 82.3% and 81.8% of joint arthroplasty and pediatric patients, respectively, indicated that using the OpenCap technology would “definitely” or “probably” reduce their burden to travel for in-person assessment, in comparison to only 68.3% of sports medicine patients (Figure 5).

For the 30.3% of respondents at this level who provided free-text justification for their indicated comfort levels, reasons for reduced comfort with these tasks included feeling uncomfortable recording themselves, anticipated increases in difficulty or pain while performing the indicated functional activities, unfamiliarity with the OpenCap technology, potential medical record concerns, and technological challenges. For the 24.7% of respondents at this level who provided free-text justification for their perceived burden reduction, living close to clinic locations and preferring in-person appointments were common reasons for indicating a lower reduction in travel burden associated with in-person evaluation.

### Analysis of tier 3 responses by residential location and household income

**Figure 6:**
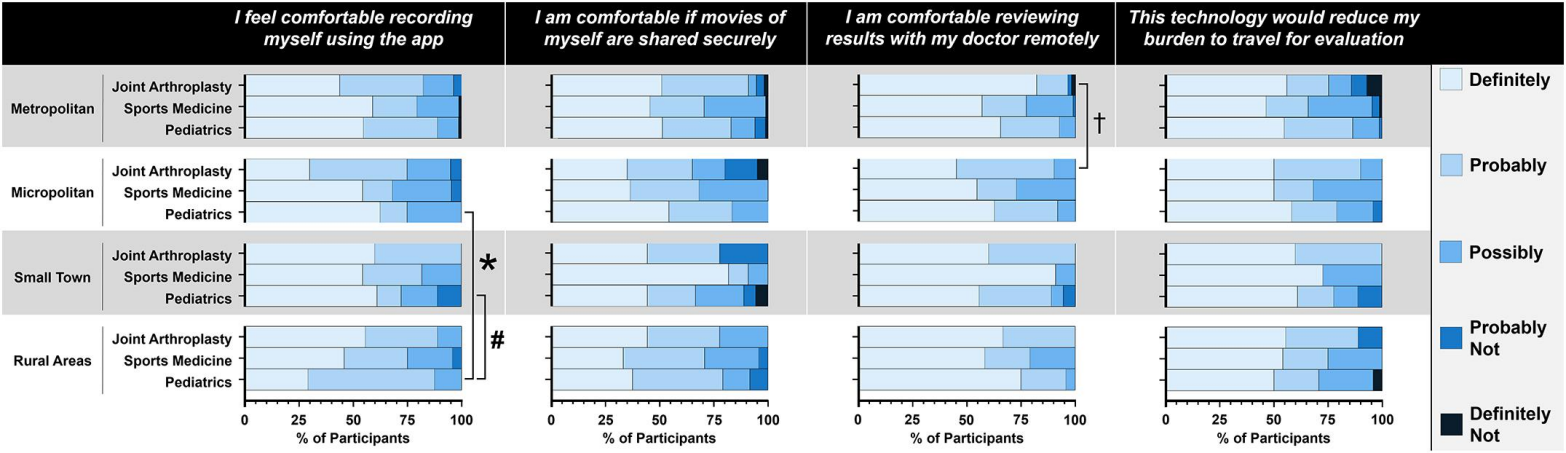
Geographic residential location-based distributions of patient comfort level performing specific tasks required to use the OpenCap technology and perceived reduction in travel burden associated with in-person evaluation. Percentages are calculated based on the total number of patients in each residential location who responded to tier-3 questions (total respondent numbers provided in third column of Table 1). Few significant differences in distributions of patient comfort level and perceived burden reduction were identified between geographic residential locations (metropolitan, micropolitan, small town, rural areas) for any of the three patient cohorts (pediatric, sports medicine, joint arthroplasty). *p = 0.027 between pediatric micropolitan and pediatric rural areas. ^#^*p* = 0.050 between pediatric small town and pediatric rural areas. ^†^*p* = 0.005 between joint arthroplasty metropolitan and joint arthroplasty micropolitan.

**Figure 7:**
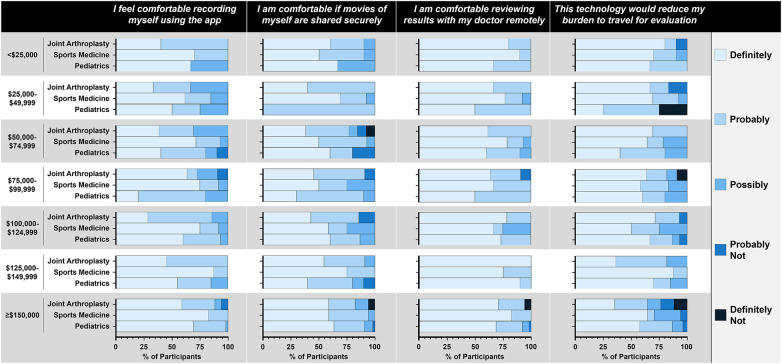
Annual household income-based distributions of patient comfort level performing specific tasks required to use the OpenCap technology and perceived reduction in travel burden associated with in-person evaluation. Percentages are calculated based on the total number of patients in each income range who responded to tier-3 questions (total respondent numbers provided in third column of Table 2). Income did not significantly affect patient comfort level recording themselves (albeit close *p* ≥ 0.051), sharing those video recordings (*p* ≥ 0.092), reviewing results remotely (*p* ≥ 0.381), or perceived burden reduction (*p* ≥ 0.394) for any of the three patient cohorts.

Neither residential location (Figure 3) nor annual household income (Figure 4) significantly affected patient-perceived ability to set up calibration equipment (residential – *p* ≥ 0.134; income – *p* ≥ 0.157) for any patient cohort. Residential location (Figure 6) only significantly affected the distribution of pediatric patient comfort level recording themselves (*p* = 0.027, micropolitan vs. rural area; *p* = 0.050, small town vs. rural area) and the distribution of joint arthroplasty patient comfort level reviewing results with their doctor remotely (*p* = 0.005, metropolitan vs. micropolitan). Annual household income (Figure 7) did not significantly affect patient comfort level recording themselves (albeit close *p* ≥ 0.051), sharing those video recordings (*p* ≥ 0.092), reviewing results remotely (*p* ≥ 0.381), or perceived burden reduction (*p* ≥ 0.394) for any of the three patient cohorts.

## Discussion

Existing studies of OpenCap, an open-source research platform for estimating 3D joint kinematics from videos captured with two iOS devices, have demonstrated its validity for musculoskeletal biomechanics assessment (21, 26–32). Yet, it is unclear whether patients presenting for orthopedic care in a predominantly rural state have adequate access to the multiple iOS devices required for an OpenCap data collection, and whether the technological burden of using a primarily research-focused platform is a barrier to its widespread implementation for remote rehabilitation management.

From the perspective of digital health adoption, our findings suggest that future integration of OpenCap as a resource for orthopedic care may be possible. Of the orthopedic patients enrolled in this study, 65.5–83.5% indicated access to the necessary technology resources for performing an OpenCap data collection (which was not significantly affected by residential location), and 71.7%–95.8% of patients indicated being “definitely” or “probably” comfortable performing activities needed for an OpenCap data collection. These findings align with existing technology adoption models, such as the Technology Acceptance Model (42, 43) and the Unified Theory of Acceptance and Use of Technology (44, 45), which suggest that key drivers of technological adoption are perceived ease of use and perceived usefulness. Further, patients perceived OpenCap as potentially being capable of alleviating travel-related burdens (68.3%–82.3% of patients felt that the OpenCap technology would “definitely” or “probably” reduce their travel burden associated with in-person evaluation). These findings highlight the patient-perceived acceptability and feasibility of future OpenCap implementation for remote rehabilitation monitoring, particularly in rural or resource-limited settings in which getting to a clinic for orthopedic care and rehabilitation management is most difficult.

Even though residential location did not significantly affect reported technological access, previous digital divide research emphasizes that device access alone does not fully capture obstacles to digital health engagement (46). Various aspects of digital health literacy, including internet quality/bandwidth, prior telehealth experience, frequency of smartphone use, caregiver assistance, operating system familiarity, technological troubleshooting ability, and disability-related usability barriers, may impact a participant's ability to successfully perform an OpenCap data collection, but these aspects were not assessed as part of this work. As such, the relatively high levels of reported device access and participant comfort level performing activities required for an OpenCap data collection may not fully generalize to populations with lower socioeconomic status, limited digital literacy, or additional accessibility needs.

While participants' reported comfort with performing tasks required for performing an OpenCap data collection suggests potential usability, this study assessed *perceived* rather than *observed* usability or clinical effectiveness. Telehealth usability frameworks such as the Nonadoption, Abandonment, Scale-up, Spread, and Sustainability (NASSS) model (47) indicate that technologies perceived as feasible may still encounter challenges to real-world implementation, such as user burden or system complexity. OpenCap's current design as a research-focused platform may require improved user-centered design and streamlined workflows for clinical integration, as well as rigorous validation of its clinical performance and impact, to ensure feasibility and efficacy for patients, clinical providers, and institutions.

Some study participants noted concerns regarding video sharing and medical record access, underscoring important privacy and data security concerns surrounding clinical implementation of technologies such as OpenCap. Potential use of cloud-based biomechanical analysis of subject-specific video data introduces important concerns related to data storage/transmission, unauthorized access, and secondary use of protected health information. The developers at Stanford University have ensured that OpenCap is compliant with the Health Insurance Portability and Accountability Act (HIPAA) and Stanford-specific requirements for cloud computing of high-risk video data, such as encrypting the data in transit and at rest, limiting data accessibility, and implementing two-factor authentication and 20-character passwords for account protection (21). Nonetheless, addressing patient, clinical provider, and institutional concerns regarding how musculoskeletal biomechanics assessment platforms like OpenCap store, process, and protect high-risk data will be critical to successful future integration in clinical practice.

There are several limitations related to measurement and study design that are worth addressing. Participants were not asked to complete an OpenCap data collection; rather, they simply responded to theoretical questions regarding technological access and perceived comfort levels with the process. This reliance on self-reported data introduces potential for overestimation of technological competence, as well as self-reporting and social desirability biases. In addition, since the study aimed only to evaluate patient-perceived feasibility of OpenCap use, the questionnaire was not formally validated, which may limit the reliability and interpretability of the study findings. Furthermore, participant perceptions also may not fully reflect real-world implementation challenges. Practical factors, such as ensuring proper camera positioning and calibration, synchronization quality, lighting conditions, space for movement, clothing, and adherence to provided data collection instructions, are crucial for successful OpenCap use, and may present barriers not captured in survey responses.

Limitations related to missing data and analytic approach should also be addressed. This study was designed to assess the most basic feasibility elements of OpenCap use across orthopedic populations in a predominately rural setting and therefore relied primarily on descriptive and univariate analyses. Future studies with larger samples and more diverse populations should employ multivariable modeling to evaluate the independent contributions of demographic, socioeconomic, geographic, and clinical factors to technological access and usability. Annual household income data were incomplete due to optional survey response, resulting in reduced sample sizes for income-based analyses. This may have decreased statistical power to detect associations, particularly in the sports medicine cohort. Nonresponse bias may have influenced income-based analyses as evidenced by statistically significant differences in some demographic comparisons between participants providing income information and those that did not (Supplementary Appendix B). Findings related to income should be interpreted with this context in mind. Additionally, the study questionnaire emphasized participant access to iOS devices due to current OpenCap platform requirements, which may further constrain interpretation; however, as the underlying technology continues to evolve, broader OpenCap compatibility with Android or other platforms may become feasible.

Finally, limitations related to generalizability and external validity are also important to discuss. The study population was drawn from a predominantly white orthopedic patient population from a single rural midwestern academic medical center. Rural academic medical centers care for a disproportionate volume of Medicaid/Medicare, unreimbursed, trauma, medically complex, and transfer patients that are vulnerable to increased health risks and poor outcomes, and reaching vulnerable patients in the most isolated, rural regions remains challenging (48). This patient population differs vastly from the orthopedic patient community at larger institutions, particularly institutions in densely populated, urban regions, and may also not be reflective of individuals who are unwilling, unable, or do not need to be seen at a tertiary care center. Differences in socioeconomic factors, racial/ethnic composition, and access to healthcare may influence variability in orthopedic condition prevalence, management, and outcomes, potentially limiting the widespread generalizability of these study findings. Future studies should evaluate more diverse patient populations from multiple geographic regions to validate the findings of this work. Furthermore, while OpenCap has been shown to be a valid musculoskeletal biomechanics assessment tool in able-bodied young adults (21, 26–28), these individuals are not representative of most orthopedic patients with deformities and/or movement abnormalities who would derive maximum benefit from such technologies. Future work investigating the validity of OpenCap for these patients, both as compared to in-gait-laboratory and in remote self-collection settings, is also warranted.

Overall, these findings suggest the patient-perceived feasibility of implementing OpenCap as a future remote musculoskeletal biomechanics assessment tool, including among orthopedic patients in predominantly rural settings. Trialing the OpenCap technology as a remote biomechanics assessment tool for specific patient populations may identify barriers to widespread implementation and determine if adding such technology to outreach services and mobile clinics (49–53) could augment the potential utility of OpenCap as a future resource for orthopedic care.

## Data Availability

The raw data supporting the conclusions of this article will be made available by the authors, without undue reservation.
