# Supplementary material for: Patient-Perceived feasibility of implementing OpenCap smartphone-based motion capture technology to remotely assess musculoskeletal movement in individuals with orthopedic disorders presenting to a rural midwestern tertiary center
Source: Front Digit Health. 2026 Jul 23;8:1870758. doi: 10.3389/fdgth.2026.1870758 (PMC13442442; doi:10.3389/fdgth.2026.1870758)
Supplement: Supplementary file 1 [file Datasheet1.docx]

**Supplementary Appendix A**

*Questionnaire on Feasibility of Using OpenCap in Orthopedic Populations*

1. Please select the provider you are seeing today:
   a. Orthopedic Surgeon A
   b. Orthopedic Surgeon B
   c. Orthopedic Surgeon C
   d. Orthopedic Surgeon D
   e. Orthopedic Surgeon E
   f. Orthopedic Surgeon F
   g. Orthopedic Surgeon G
2. Please select the orthopedic region you are being treated for (check all that apply):
   a. Spine
   b. Shoulder
   c. Elbow
   d. Wrist/Hand
   e. Hip
   f. Knee
   g. Foot/Ankle
3. Please provide more information on the orthopedic condition you are currently being treated for:
4. What is your total annual household income?
   a. Less than $25,000
   b. $25,000-$49,999
   c. $50,000-$74,999
   d. $75,000-$99,999
   e. $100,000-$124,999
   f. $125,000-$149,999
   g. $150,000 or more
   h. Prefer not to answer
5. Would you be able to have access to two iOS devices (iPhone or iPad) simultaneously? (Combination of family, friends, neighbors)

____ Yes	____ No

**If answer to Question 5 is Yes**:

6. Would you be able to set up the two iOS devices in a manner that keeps them stationary for recording? (e.g. on a tripod or stably balanced)

____ Yes	____ No

7. At the time you could access 2 iOS devices, could you also have access to a computer with an Internet connection?

____ Yes	____ No

8. Are you comfortable installing and using an app on the two iOS devices that accesses the device’s camera?

____ Yes	____ No

**If answers to Questions 6-8 are Yes:**

9. Would you be able to set up a calibration object (checkerboard on plexiglass sheet provided to you) so that it is perpendicular to the ground (for example, up against a wall) in the same location where you are recording?

____ Yes	____ No

10. I would feel comfortable recording myself walking, squatting, and performing a sit-to-stand task using the app on the two iOS devices.

____Definitely	____Probably	____Possibly	____Probably Not	____Definitely Not

Please describe why you selected your answer:

11. I would feel comfortable for movies of myself to be shared securely to a computer server at Stanford University for automated analysis?

____Definitely	____Probably	____Possibly	____Probably Not	____Definitely Not

Please describe why you selected your answer:

12. I would be comfortable reviewing results from the analysis with my doctor remotely?

____Definitely	____Probably	____Possibly	____Probably Not	____Definitely Not

Please describe why you selected your answer:

13. Being able to provide information to my doctors and therapists would reduce the burden on me to return to clinic for evaluation.

____Definitely	____Probably	____Possibly	____Probably Not	____Definitely Not

Please describe why you selected your answer:

14. What concerns do you have about doing this analysis yourself from your home?

**Supplementary Appendix B**

|  | *Pediatrics*  *No Income*  *Provided*  *(n = 47)* | *Pediatrics*  *Income*  *Provided*  *(n = 153)* | *p-value* | *Sports Medicine*  *No Income*  *Provided*  *(n = 84)* | *Sports Medicine*  *Income*  *Provided*  *(n = 116)* | *p-value* | *Joint Arthroplasty*  *No Income*  *Provided*  *(n = 44)* | *Joint Arthroplasty*  *Income*  *Provided*  *(n = 156)* | *p-value* |
| --- | --- | --- | --- | --- | --- | --- | --- | --- | --- |
| *Age, years (Median [IQR])* | 14 [12-16] | 14 [12-15] | 0.425 | 26.5 [21.8-37] | 29 [21-39] | 0.633 | 72 [64.8-77] | 65.5 [58-71] | **<0.001** |
| *Female (n [%])* | 23 [48.9] | 82 [53.6] | 0.576 | 51 [60.7] | 76 [65.5] | 0.552 | 24 [54.5] | 90 [57.7] | 0.710 |
| *Race* |  |  | 0.426 |  |  | 0.309 |  |  | 0.667 |
| *American Indian or*  *Alaska Native (n [%])* | 0 [0] | 1 [0.7] |  | 0 [0] | 0 [0] |  | 0 [0] | 1 [0.6] |  |
| *Asian (n [%])* | 1 [2.1] | 0 [0] |  | 1 [1.2] | 0 [0] |  | 1 [2.3] | 3 [1.9] |  |
| *Black or African American (n [%])* | 1 [2.1] | 7 [4.6] |  | 3 [3.6] | 4 [3.4] |  | 3 [6.8] | 5 [3.2] |  |
| *Native Hawaiian or*  *Other Pacific Islander (n [%])* | 0 [0] | 0 [0] |  | 0 [0] | 1 [0.9] |  | 0 [0] | 1 [0.6] |  |
| *Unknown (n [%])* | 5 [10.6] | 12 [7.8] |  | 9 [10.7] | 6 [5.2] |  | 3 [6.8] | 6 [3.8] |  |
| *White (n [%])* | 40 [85.1] | 133 [86.9] |  | 71 [84.5] | 105 [90.5] |  | 37 [84.1] | 140 [89.7] |  |
| *Ethnicity* |  |  | 1.000 |  |  | 0.437 |  |  | 0.700 |
| *Hispanic or Latino (n [%])* | 1 [2.1] | 4 [2.6] |  | 2 [2.4] | 3 [2.6] |  | 0 [0] | 1 [0.6] |  |
| *Non-Hispanic (n [%])* | 45 [95.7] | 143 [93.5] |  | 76 [90.5] | 109 [94] |  | 42 [95.5] | 151 [96.8] |  |
| *Unknown (n [%])* | 1 [2.1] | 6 [3.9] |  | 6 [7.1] | 4 [3.4] |  | 2 [4.5] | 4 [2.6] |  |
| *Orthopedic Condition Being Treated* |  |  | **0.006** |  |  | 0.701 |  |  | 0.229 |
| *Elbow (n [%])* | 2 [4.3] | 15 [9.8] |  | 0 [0] | 1 [0.9] |  | 0 [0] | 0 [0] |  |
| *Foot/Ankle (n [%])* | 11 [23.4] | 13 [8.5] |  | 0 [0] | 0 [0] |  | 0 [0] | 0 [0] |  |
| *Hip (n [%])* | 0 [0] | 14 [9.2] |  | 41 [48.8] | 60 [51.7] |  | 27 [61.4] | 77 [49.4] |  |
| *Knee (n [%])* | 9 [19.1] | 12 [7.8] |  | 36 [42.9] | 46 [39.7] |  | 17 [38.6] | 82 [52.6] |  |
| *Shoulder (n [%])* | 0 [0] | 4 [2.6] |  | 6 [7.1] | 12 [10.3] |  | 0 [0] | 0 [0] |  |
| *Spine (n [%])* | 24 [51.1] | 91 [59.5] |  | 1 [1.2] | 0 [0] |  | 0 [0] | 2 [1.3] |  |
| *Wrist/Hand (n [%])* | 4 [8.5] | 14 [9.2] |  | 0 [0] | 0 [0] |  | 0 [0] | 0 [0] |  |
| *Residential RUCA Classification* |  |  | 0.771 |  |  | 0.999 |  |  | 0.514 |
| *Metropolitan (RUCA 1-3) (n [%])* | 24 [51.1] | 86 [56.2] |  | 50 [59.5] | 70 [60.3] |  | 28 [63.6] | 103 [66.0] |  |
| *Micropolitan (RUCA 4-6) (n [%])* | 8 [17] | 23 [15] |  | 12 [14.3] | 16 [13.8] |  | 5 [11.4] | 23 [14.7] |  |
| *Small Town (RUCA 7-9) (n [%])* | 8 [17] | 18 [11.8] |  | 8 [9.5] | 11 [9.5] |  | 3 [6.8] | 14 [9.0] |  |
| *Rural Area (RUCA 10) (n [%])* | 7 [14.9] | 26 [17] |  | 14 [16.7] | 19 [16.4] |  | 8 [18.2] | 16 [10.3] |  |

**Table 2:** Comparison of demographic characteristics and orthopedic condition being treated in pediatric, sports medicine, and joint arthroplasty patients between those that provided annual household income and those that did not.
